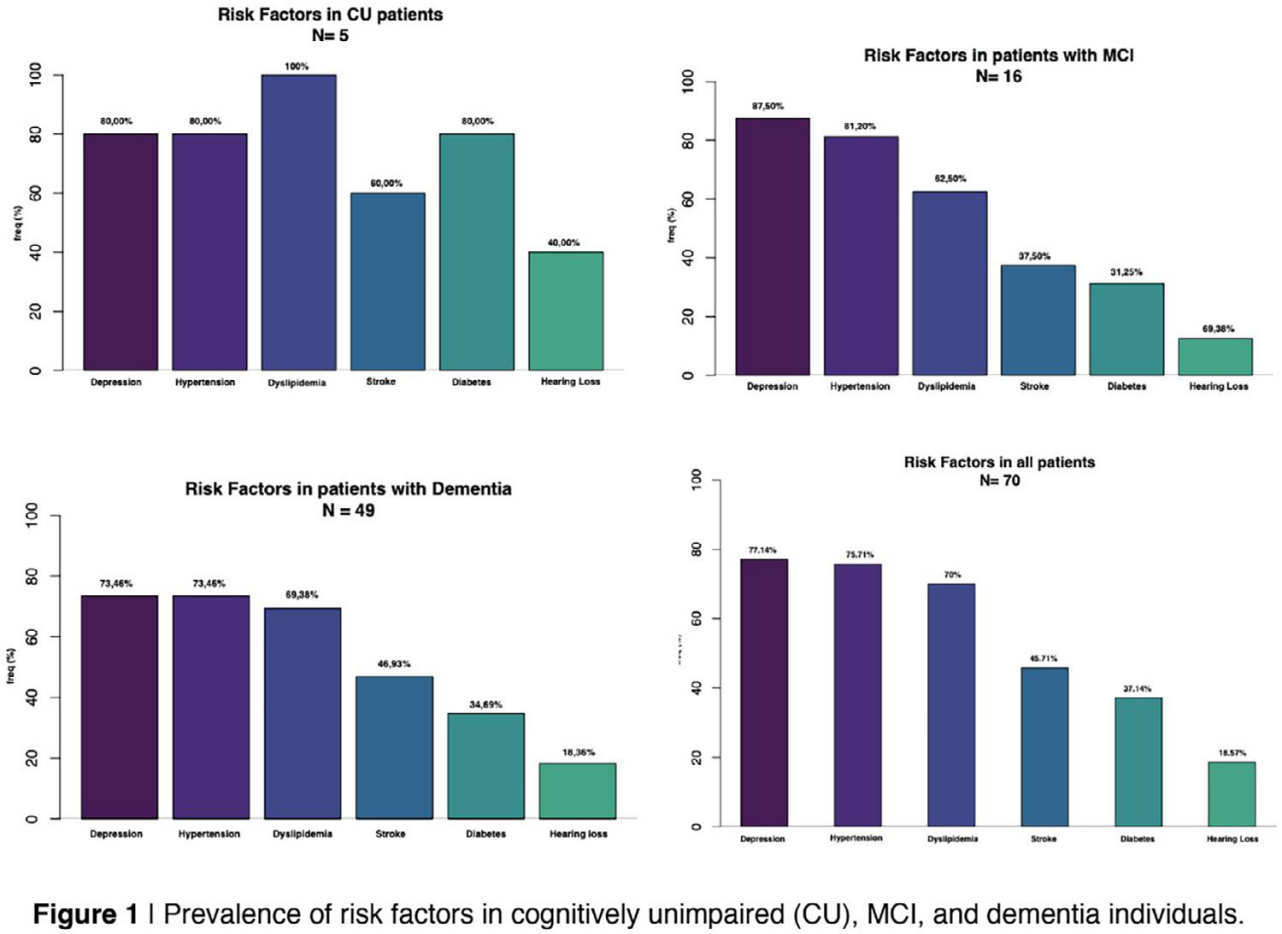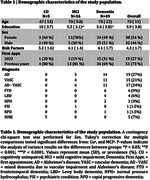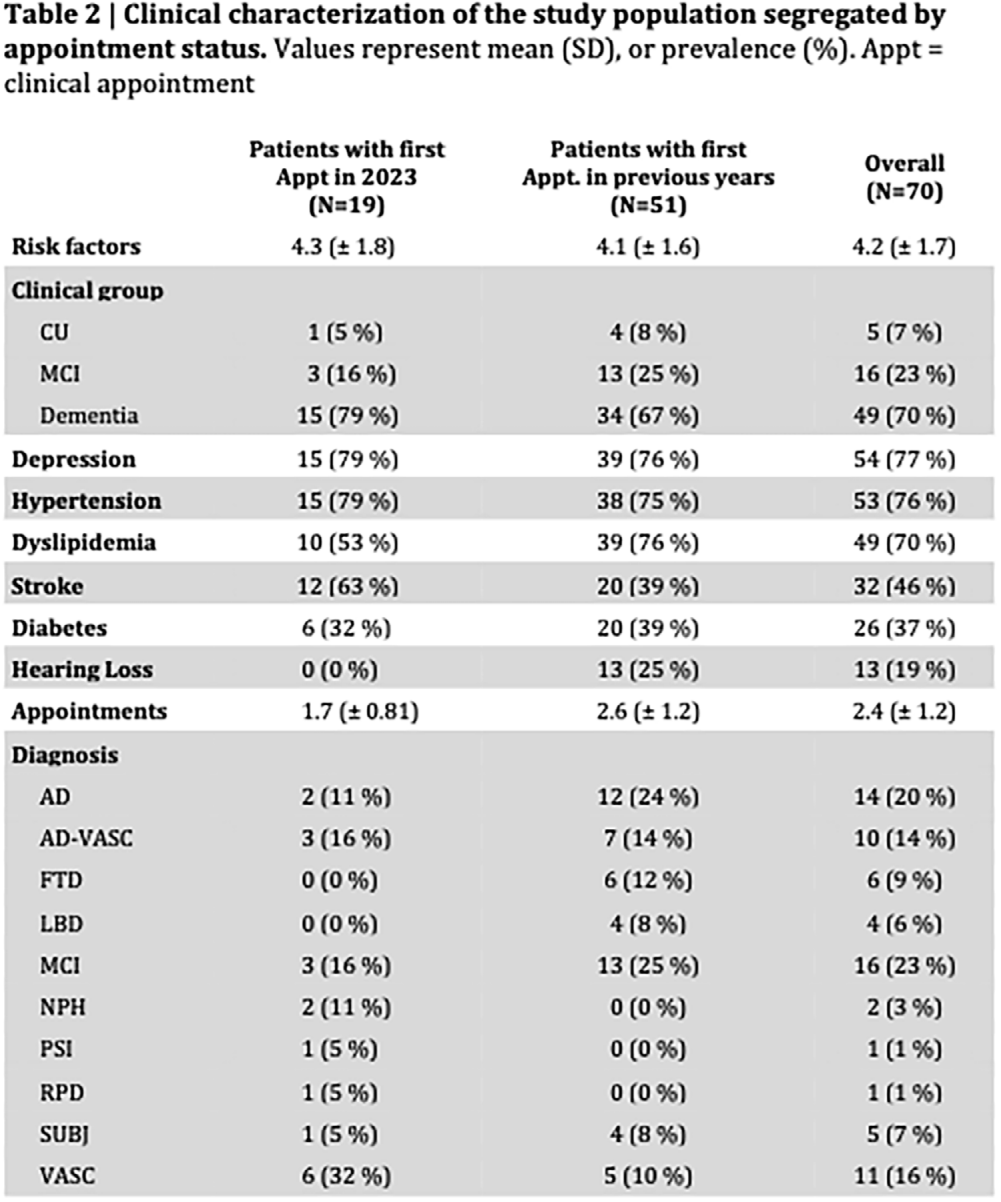# Modifiable dementia risk factors in a memory outpatient clinic in Brazil

**DOI:** 10.1002/alz.092894

**Published:** 2025-01-09

**Authors:** Fernando Jacob Lazzaretti, Andressa de Oliveira Felício, Julia Patatt, Cristiano Schaffer Aguzzoli, Lucas Porcello Schilling

**Affiliations:** ^1^ PUCRS, Porto Alegre, Rio Grande do Sul Brazil; ^2^ Brain Institute of Rio Grande do Sul, PUCRS, Porto Alegre, RS Brazil; ^3^ Global Brain Health Institute, San Francisco, CA USA

## Abstract

**Background:**

Low and middle income countries (LMIC) present a higher prevalence of dementia compared to high‐income countries due to health and social‐related modifiable risk factors inequities. Albeit the clear identification of these risk factors, the assessment and management of those situations before the development of dementia lacks attention in different health care levels. Thus, we aim to investigate the clinical impact and prevalence of health‐related risk factors among individuals referred to a specialized tertiary outpatient memory clinic from primary care facilities.

**Method:**

We assessed 70 individuals (5 cognitively unimpaired (CU), 16 mild cognitive impairment (MCI), and 49 dementia) from an Brazilian Unified Public Health outpatient memory clinic from São Lucas Hospital located in the south of Brazil. The patients underwent clinical assessments comprising cognitive evaluation and a full neurologic examination. All participants underwent magnetic resonance imaging (MRI) and the clinical diagnosis was established by two experienced cognitive neurologists. We estimated the prevalence of risk factors for mild cognitive impairment (MCI) and dementia following those proposed by the Lancet Commission 2020. Tukey’s correction for multiple comparisons tested significant differences in risk factors among clinical groups.

**Result:**

Mean age of the study population was 73 years (range, 31‐90 years); and 46% of whom were male. The most prevalent risk factors were depression (77.14%), hypertension (75.71%) and dyslipidemia (70%). Other notable factors included stroke (45.71%), diabetes (37.14%) and hearing loss (18.57%) (Figure 1). CU patients had significantly higher education attainment compared to MCI and dementia groups (Table 1). Stratifying the population according to appointment status revealed that patients who had their first appointment in 2023 exhibited a higher prevalence of depression (79%), hypertension (79%) and stroke (63%) (Table 2).

**Conclusion:**

Our results highlight the high prevalence of dementia risk factors in patients referred by primary care units, but also in the outpatient memory clinic. Our findings provide a rationale for an urgent need of a better assessment and treatment of risk factors to reduce dementia incidence in LMIC.